# An Update on Treatment Options for Methicillin-Resistant Staphylococcus aureus (MRSA) Bacteremia: A Systematic Review

**DOI:** 10.7759/cureus.31486

**Published:** 2022-11-14

**Authors:** Fatema Mahjabeen, Utsow Saha, Mayesha N Mostafa, Farzana Siddique, Eram Ahsan, Sawsan Fathma, Anika Tasnim, Tasnim Rahman, Ridwan Faruq, Md Sakibuzzaman, Fahmida Dilnaz, Adrita Ashraf

**Affiliations:** 1 Cardiology, National Institute of Cardiovascular Diseases, Dhaka, BGD; 2 Medical School, Enam Medical College & Hospital, Rangpur, BGD; 3 Internal Medicine, Delta Medical College, Dhaka, BGD; 4 Internal Medicine, Sir Salimullah Medical College, Dhaka, BGD; 5 Research and Development, Mayo Clinic, Rochester, USA; 6 Anesthesiology, Mayo Clinic, Rochester, USA; 7 Internal Medicine, Bangladesh Medical College and Hospital, Dhaka, BGD; 8 Internal Medicine, Brookdale University Hospital Medical Center, New York City, USA; 9 Medical School, Wenzhou Medical University, Wenzhou, CHN; 10 Internal Medicine, Yale New Haven Bridgeport Hospital, Bridgeport, USA; 11 Neurology, University of Toledo, Toledo, USA; 12 Internal Medicine, University of Mississippi Medical Center, Jackson, USA; 13 Experimental Pathology (Cancer Biology), Mayo Clinic, Rochester, USA; 14 Internal Medicine, Jalalabad Ragib Rabeya Medical College, Sylhet, BGD; 15 Internal Medicine, Northeastern Health System Hospital, Tahlequah, USA

**Keywords:** staphylococcus aureus, ceftaroline, daptomycin, vancomycin, bacteremia, mrsa

## Abstract

Since the last century, methicillin-resistant *Staphylococcus aureus* (MRSA) bacteremia has become a major global and public health concern not only in terms of morbidity and mortality but also the duration of hospital stay, healthcare cost, and antimicrobial choices. Especially alarming is the growing antimicrobial resistance due to their misuse and overuse, which has led the world to be exhausted of its effective antibiotic resources. In this review article, we sought to figure out the most efficacious antimicrobial agents to treat MRSA-related bloodstream infections. We compared the data from reviewing reports from 2017 to 2022 and summarized their comparative efficacy and cost-effectiveness. Although we focused on vancomycin and daptomycin, which are the current Infectious Disease Society Of America (IDSA)-recommended antibiotics for MRSA bacteremia treatment, a deep dive into the newer agents revealed better efficacy and treatment outcome in the combination of ceftaroline (β-lactam) with daptomycin compared to traditional standard monotherapy (vancomycin/daptomycin monotherapy). Also, the IDSA recommended high-dose daptomycin (8-10 mg/kg) therapy for MRSA bacteremia treatment to be more effective in cases with vancomycin-reduced susceptibility. Moreover, we did not find any trial or study describing the use of ceftaroline as a monotherapy to compare its efficacy in MRSA bacteremia with the current standard therapy. The upshot is that we need more large-scale clinical trials exploring in-depth effectiveness and adverse effects to decide on newer agents like β-lactams to use as routine therapy for MRSA bacteremia.

## Introduction and background

In 1960, isolates of S*taphylococcus aureus* resistant to methicillin were discovered [1]. Today, methicillin-resistant* Staphylococcus aureus* (MRSA) is a significant menace to healthcare, with 25% of the S*taphylococcus aureus* isolates showing methicillin resistance in several countries, including the United States, over the last decade [2]. Although for some time, MRSA was only thought to occur in healthcare settings, the scenario became more problematic from the mid-1990s onwards as MRSA infections started to emerge increasingly in individuals who were not exposed to the healthcare system [3].

Owing to the plethora of virulence factors and its adaptability in new milieus,* Staphylococcus aureus* can cause many different infections, ultimately leading to bacteremia [4]. Furthermore, the dissemination of the organism into the bloodstream leads to grave conditions such as osteomyelitis, endocarditis, and sepsis [5]. Earlier studies have demonstrated that mortality rates due to MRSA reached as high as 60%. The Centers for Disease Control and Prevention (CDC) lists 80,000 cases of invasive infections and 11,000 deaths yearly attributable to MRSA [6]. These concerning figures for morbidity and mortality are further compounded by the prolonged treatment durations and sky-high healthcare costs, which are increasing, as found by a retrospective study that used data from 2010-2014 [2]. According to the CDC, annual healthcare costs total up to $3-4 billion in the US alone [7]. Although there has been a decline in prevalence in the US (demonstrated by data from 2013-2016), the high mortality rates continue to make MRSA an ongoing threat.

Current guidelines governed by the Infectious Diseases Society of America (IDSA) recommend vancomycin (VAN) or daptomycin (DAP) as first-line agents for treating MRSA bacteremia (MRSA). While both have proved to be efficacious, neither is without limitations [6]. A promising alternative to these regimens is ceftaroline, a β-lactam antibiotic, which has been studied in combination with DAP and VAN. The IDSA guidelines are being updated, and it is crucial to recognize how drastically guidelines direct clinician practices [8].

The lack of high-quality head-to-head comparative studies demonstrating the role of these antimicrobials in the treatment [6] of MRSAB makes it challenging to deal with such cases effectively. Furthermore, there have only been three studies (including exploratory research) analyzing the cost-effectiveness of multiple agents used in clinical practice [8], which poses a problem as it is essential to incorporate the cost and health benefits in a modern healthcare system [9].

Given the clinical burden of MRSA bacteremia, clinicians need to have a comprehensive idea of the different treatment options available, both old and new, including the mechanism of resistance, efficacy, safety profiles, and cost-effectiveness. In this systematic review, we aim to extensively compare and contrast the available options for MRSA bacteremia so that clinicians can employ an all-encompassing approach while prescribing the most appropriate antibiotic that suits particular patient profiles.

## Review

Method

Information Sources and Keyword Search

The following items were used in PubMed searches to find relevant articles: (treatment of MRSA bacteremia), (methicillin-resistant *Staphylococcus aureus* or MRSA), (MRSA bacteremia). In addition, the reference lists of reports identified by this search strategy were also searched to select relevant articles.

Inclusion and Exclusion Criteria

Review articles were selected and reviewed. Studies involving Adult patients with MRSA bacteremia were included. Articles of MRSA bacteremia and treatment with different antibiotics were included and reviewed. Standard therapy treatment (VAN or DAP alone treatment) and standard therapy combination with β-lactams were compared. Animal and in vitro studies and studies involving the pediatric population was excluded.

Data Collection and Extraction

**Figure 1:**
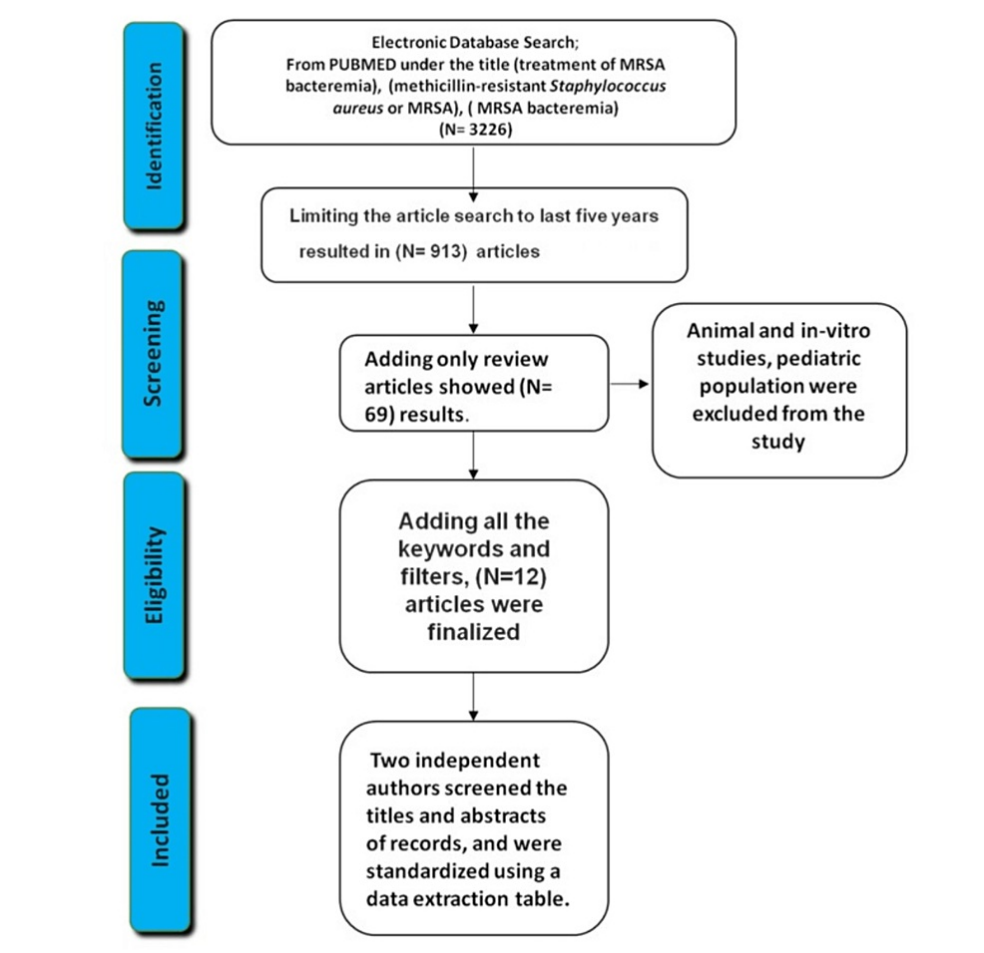
PRISMA flow diagram of the included studies. PRISMA: Preferred Reporting Items for Systematic Reviews and Meta-Analyses; MRSA: methicillin-resistant *Staphylococcus aureus*

An initial search on PubMed for MRSA bacteremia results showed 3226 articles (Figure 1). Literature searches were limited to articles published from 2017 to 2022. This resulted in 913 pieces and including only review articles showed 69 results. After adding all the keywords and filters, 12 papers were finalized. To analyze possibly eligible reports, two independent authors screened the titles and abstracts of records, which were standardized using a data extraction table.

Comparative efficacy of VAN, DAP, and β-lactam

When discussing the management of bacteremia, it is essential to differentiate between complicated and uncomplicated bacteremia as it guides the direction. Bacteriemia is involved if a patient has positive blood culture for MRSA with the presence of any of the following: endocarditis, implanted prosthesis, metastatic infection, blood culture done two to four days after the initial culture grows MRSA, persistent fever after 72 hours of commencing therapy [10].

The choices of antibiotics approved to treat MRSA bacteremia by the IDSA currently are VAN and DAP [1]. However, each agent has its own set of regulations. VAN is the initial choice in MRSA bacteria and should be optimally dosed.

Keeping in mind the importance of preventing the emanation of drug resistance while maintaining significant efficacy with most minor side effects compared to the existing antibiotics, the IDSA has commended high-dose DAP (8-10 mg/kg) for complicated MRSA bacteremia. The Cubicin Outcomes Registry and Experience (CORE; US) and European Cubicin Outcomes Registry and Experience (EU-CORE; Europe, Latin America, and Asia) involving treatment of serious MRSA infections like bacteremia, infective endocarditis (IE), and osteomyelitis also used this high-dose regimen following the recommendation [11].

This higher dosage of DAP has not been reliably proven to prevent its nonsusceptibility. The US Food and Drug Administration (FDA) has recommended 6 mg/kg/day for MRSA bacteremia [12,13].

Several national and international studies provide data regarding the benefits of a higher dosage of DAP and its pitfalls. A nationwide retrospective cohort study comprising 371 MRSA bacteremia patients, had shown a survival benefit at 30 days when the regimen was changed to DAP within seven days of VAN therapy in those with DAP dosage more or equal to 7 mg/kg/d in comparison to 6 mg/kg/d dosing (hazard ratio (HR) 0.31 with 95%CI 0.10-0.94) [14].

Murray et al. described the cases of 85 MRSA bacteremic patients who were changed to DAP median dose of 8.4 mg/kg/day after a median of 1.7 days of VAN therapy where VAN minimal inhibitory concentration (MIC) was more or equal to 1.5 mg/dL. They were compared with the 85 matched counterparts who only received VAN (median trough 17.6 mg/mL). The results were both fewer treatment failures (20% vs. 48.2%, p<0.001) and 30-day mortality (3.5% vs. 12.9%, p=0.047) compared to VAN [15]. Similar results were found in a separate study conducted by Claeys and colleagues, where 51.9% of patients received DAP at a dose of more or equal to 8 mg/kg/d, irrespective of VAN MIC [16]. Another retrospective analysis revealed no significant improvement in the composite failure rate (all-cause mortality at 60 days, clinical or microbiological failure at seven days, BSI relapse at 30 days, or end-of-treatment failure (EOT; discontinuation or change of daptomycin because of therapeutic failure or adverse reaction)) having an early transition to DAP but the enhanced risk of nephrotoxicity in prolonging VAN therapy [17].

Unsusceptible isolates and treatment failures have been reported despite DAP's effectiveness against MRSA bacteremia. Due to the increasing number of cases of resistance or therapeutic failure to first-line antibiotics, physicians have started to look for other alternatives. Among these alternatives, β-lactams, especially ceftaroline, have shown promise to be an integral part of the future MRSA treatment protocol. Many in vitro studies proved that β-lactam drugs are effective for treating MRSA resistant to VAN or DAP [18,19]. These in vitro studies show that β-lactam efficacy increases as MRSA susceptibility to VAN or DAP decreases. This phenomenon is called the “seesaw effect.” Ceftaroline reduces cell wall cross-linking and thickness and allows DAP to access the cell membrane easily. DAP disrupts cell membrane integrity; therefore, combining it with ceftaroline enhances its bactericidal activity against MRSA [20]. Based on the in vitro studies, various studies have been performed to see whether β-lactams are eligible to be used in MRSA bacteremia in clinical settings.

The retrospective studies by Dilworth et al. [21] and Casapao et al. [22] compare VAN monotherapy with β-lactam VAN combination treatments. These studies show that the duration of bacteremia reduces with combination treatment. In the study by Dilworth et al., patients receiving the combination treatment were 11.24 (95% CI, 1.7 to 144.3 times; P = 0.01) times more likely to achieve bacterial eradication (defined as negative blood culture with no relapse in 30 days) than patients receiving VAN alone. But these studies could not provide reliable data on clinical success rates and focused only on bacterial clearance.

Davis et al. performed the Combination Antibiotics for Methicillin-Resistant *S.aureus* (CAMERA) study, an open-label multicenter randomized control trial of patients with MRSA where 31 patients received VAN with flucloxacillin and 29 received only VAN [23]. The mean duration of bacteremia in patients receiving combination treatment was 1.79 days, and in patients receiving VAN monotherapy was three days. There was no change in rates of 28-day or 90-day mortality. This study also noted a higher incidence of nephrotoxicity in patients receiving combination therapy, but it was not statistically significant.

Tong et al. published a CAMERA2 study that randomized 352 patients with MRSA bacteremia to standard therapy (VAN or DAP) or combination therapy with β-lactams (VAN/DAP with flucloxacillin/cloxacillin) [24]. This study was done to see the effect of the addition of 7 days of antistaphylococcal β-lactam to standard therapy of MRSA treatment, and the results failed to show any statistically significant change in the duration of bacteremia or mortality rate as the trial was stopped early due to increased incidence of acute kidney injury in patients receiving combination therapy.

In MRSA bacteremia or endocarditis, a recent meta-analysis compared the clinical efficacy and safety of VAN or DAP combined BL vs. VAN or DAP monotherapy [25]. According to the studies, combination therapy had a lower risk of clinical failure (OR = 0.56; 95% CI, 0.39 to 0.79; P<0.001; I² = 26.22 %), but there was no significant difference in mortality or nephrotoxicity between the two treatment options. Another meta-analysis found that, while combination therapy reduced clinical failure, bacteremia recurrence, persistent bacteremia, and bacteremia duration, BLs as adjuvant therapy for MRSA bacteremia did not improve crude mortality compared to standard treatment [1].

A systematic review and meta-analysis in 2021 by Yi et al. compared the efficacy and safety of β-lactam combination treatment with standard monotherapy in MRSA bacteremia patients. The study included three randomized clinical trials and 10 observational studies, among which three studies compared only ceftaroline combination treatment with standard monotherapy. Even though patients treated with β-lactam showed a reduction in the duration of bacteremia, lower rate of persistent bacteremia, and lower recurrence rate, the overall study did not find any significant difference in clinical outcomes, including mortality within 30 days and 60-90 days, in-hospital mortality or length of hospital stay rate. However, a reduced 30-day mortality rate was observed for combination therapy. Most of these studies either had small populations or were terminated prematurely due to high bias. An increased risk of acute kidney injury was found in patients receiving flucloxacillin or cloxacillin [26].

A multicenter retrospective case-control study compared ceftaroline with VAN to treat MRSA after VAN failure [27]. The study chose 32 patients with VAN MIC≥ 2 μg/mL and matched 16 cases with 16 controls based on age and source of infection. Among the cases, 15 received 600mg ceftaroline every eight or 12 hours after initial treatment with VAN (and in some cases also DAP/linezolid) for a median of five days, and only one received ceftaroline initially. The controls did not receive any treatment with ceftaroline and continued to receive VAN (or DAP, linezolid, tigecycline, and rifampicin). Blood samples showed negative culture within a median of four days in the ceftaroline-treated cases compared to eight days among the controls. Thirteen out of 16 (81%) ceftaroline-treated cases were successful compared to the seven out of 16 (44%) patients in the control group. The recurrence rate was also more in the control group (38%) than in the case group (6%). However, this study was not statistically significant due to its small number of cases.

As a result, it's crucial to weigh the risks and benefits of adding a second antimicrobial drug to treat MRSA bacteremia. More randomized controlled studies focusing on combination therapy combinations, dosages, administration techniques, and treatment duration would be needed in the future to assess the evidence for the mortality and safety of combination therapy.

VAN

Mechanism of Resistance

VAN resistance is mediated by multiple genes that encode different ligases. Depending on which type of ligase the genes encode for, we can divide them into levels of resistance. Genes encoding for D-Ala: D-Lac ligase leads to alterations resulting in a high level of resistance. While genes are encoding D-Ala: D-Ser ligases lead to changes that result in a low level of resistance.

Among the different genes causing high levels of resistance (VanA, VanB, VanD, VanF, vanI, and VanM), VanA is essential as it produces isolated VAN-resistant *Staphylococcus aureus* (VRSA) strains. It encodes the enzymes that can alter or remove the binding site of VAN. It removes the D-ala -D-ala in the C- terminal and then restores it with D-alanyl-D-lactate. As a result of this change, the affinity of the binding site for VAN is significantly reduced. This indicates that we need to find other places for the binding of VAN with the help of gene sequencing [28].

Mechanism of Action

VAN antimicrobial activity is mediated through the prevention of cell wall synthesis. VAN binds to its binding site, i.e. D-Ala-D-Ala residue of C terminal, and prevents the cross bridging resulting in inhibition of peptidoglycan formation [28].

Adverse Effects

Acute Kidney Injury (AKI) is one of the most worrisome adverse effects of VAN. It has been found that the chances of VAN-induced AKI increase with the increase in trough concentrations. Trough concentrations greater than equal to 20 microgram/mL correspond to a marked increase in cases of developing AKI [29].

In a study by Tong et al., where β-lactam was combined with standard therapy, i.e with VAN or DAP for treating MRSA bacteremia, it showed that all-cause 90-day mortality was not significant (absolute difference, −4.2%; 95%CI, −14.3% to 6.0%) [24]. AKI incidence was also reported more in the combination group with a statistically significant difference of 17.2%, with 95%CI, 9.3%-25.2% [24].

DAP

Mechanism of Action

DAP follows a rapid cell death model, which is why it acts as a faster bactericidal agent than other equivalent antibiotics [30]. DAP works on the cell envelope of the bacteria. It first attaches to the cell membrane with a calcium-dependent system and disrupts the integrity of the membrane, which in turn releases intracellular ions and precipitates cell death.

Mechanism of Resistance

There is evidence of substantial genomic evolution leading to antibiotic resistance of *Staphylococcus aureus* in patients with persistent MRSA bacteremia who are under prolonged and high-dose antibiotic treatment [31]. Claeys et al., showed in a retrospective propensity-matched cohort study of 262 patients, that higher clinical failure (defined as 30-day mortality, >7 days of bacteremia, and worsening of clinical features leading to the change in anti-MRSA therapy) for patients receiving VAN compared with DAP (45.0% vs. 29.0%; P = 0.007) regardless of VAN MIC [16]. Resistance can occur through horizontal gene transfer mediated by plasmids or other mobile genetic elements or mutations in chromosomal genes [32,33]. Using the dynamics of the changing cell wall structure and cell membrane phospholipid metabolism increasing cell surface positive charge, there is the development of DAP resistance [34].

Several phospholipid components of the cell wall and cell membrane are thought to be directly related to DAP resistance. Phosphatidylglycerol (PG), lysyl-phosphatidylglycerol, and cardiolipin are among them [28]. Reduced PG production, enhanced PG conversion to lysyl-PG, and faster transport of lysyl-PG to the outer side of the cell membrane [35] result in the accumulation of positive charge on the outer surface of the cell membrane. Genes involved in resistance mechanisms are MprF, ClpP, RpoC, DltB, DltD, VraG, SpsB, FmtA, Asp23, YycFG, VraSR, and PgsA [36]. Various mutations in these genes lead to changes in cell wall phospholipid composition, increased membrane thickness and cell wall mass, and accumulation of positive cell-surface charges, which ultimately impart reduced susceptibility to DAP [37-48].

Adverse Effects

The most frequently encountered adverse effect with DAP is rhabdomyolysis [6], with rises in creatine phosphokinase (CPK) seen with a dose of 6 mg/kg/day but not lower [10]. Therefore, patients on DAP who are on concomitant statin therapy or with renal insufficiency, muscle pain, or weakness should monitor their CPK levels. There have also been some reported cases of eosinophilic pneumonitis [10].

Cost-Effectiveness

An exploratory study investigating the cost-effectiveness of several antibiotics (including DAP) in treating MRSA bacteremia concluded that DAP was more expensive but also more effective than other drugs at four and six weeks. Monitoring costs for DAP were also cut down since it is only limited to monitoring creatine phosphokinase levels in patients with myalgia. Daptomycin can also be used as outpatient antibiotic therapy (OPAT) allowing for further budget cuts [8].

Another study by Browne et al. evaluated the cost of treatment with DAP compared to VAN in a population of patients with confirmed MRSA bacteremia and infective endocarditis [9]. The study took into account only direct medical costs, i.e., drug costs, inpatient stay, monitoring tests, and outpatient care, with inpatient stay being the primary determinant. Assuming the same treatment duration, the direct medical cost per patient for DAP and VAN was found to be £17,917 and £17,165, respectively. A sensitivity analysis determined that when the treatment duration of DAP was decreased by 20%, there was a reduction in the cost of £62. Moreover, the reduced need for second-line therapies and monitoring tests as DAP has a better safety and efficacy profile than VAN further neutralizing the higher cost per vial of DAP. Taking all these factors into consideration and if the higher efficacy of DAP allows for a shorter inpatient stay, DAP can result in healthcare cost savings.

Ceftaroline (β-lactam)

Mechanism of Action

Ceftaroline, an active metabolite of the prodrug ceftaroline fosamil, is an intravenous fifth-generation cephalosporin with bactericidal activity against Gram-positive organisms, including MRSA. It was approved by the FDA in 2010 for managing acute bacterial skin and skin structure infections (ABSSSI) and community-acquired bacterial pneumonia along with concurrent bacteremia [49].

Ceftaroline stands unique compared to all other cephalosporins due to its molecular structure that results in increased binding affinity to penicillin-binding protein 2A (PBP-2A), which increases its bactericidal activity against MRSA compared to other cephalosporins [50-52].

Resistance to Ceftaroline

During a global surveillance program conducted in 2010, 8037 *S. aureus* isolates were tested for ceftaroline susceptibility. Among them, only four MRSA isolates were identified to have ceftaroline resistance with a MIC of > 2mg/dl [53]. The changes that contributed to ceftaroline MIC values were primarily in the mecA genes of MRSA isolates, which encode the PBP and PBP2a.

According to the data collected, a single amino acid alteration at crucial residue in the non-penicillin-binding domain (nPBD) of PBP2a will give rise to a slight elevation of ceftaroline MIC value where an additional substitution in the penicillin-binding-domain (PBD) is associated with a further height in the ceftaroline MIC value [53].

Adverse Effects

Increased incidence of AKI is noted in patients receiving anti-staphylococcal β-lactam and VAN/DAP combination treatment compared to those receiving standard therapy with VAN or DAP monotherapy. But, this problem did not arise in patients receiving cephalosporins [24]. A systematic review [54] in 2019 investigated the effects of cefazolin compared to antistaphylococcal β-lactams in treating MRSA bacteremia and found cefazolin to have similar efficacy with less significantly low nephrotoxic impact in the patients. Ceftaroline can predispose a patient to hypersensitivity, anaphylaxis, eosinophilia, *Clostridium difficile* infections (CDI), agranulocytosis, and leukopenia [55]. Multiple reports showed that these adverse drug reactions were rare when ceftaroline was prescribed for less than seven days. The adverse effects rate increased with the ceftaroline course's duration or higher dosage [49]. Physicians should particularly look out for agranulocytosis as recent studies have shown that almost 13% of patients treated with a prolonged course of ceftaroline (>7 days) developed agranulocytosis [56-59]. In severe cases, the neutrophil count dropped to 0 cells/mm [60], and therefore it is recommended to monitor patients prescribed prolonged courses (>21 days) of ceftaroline for leukopenia.

Cost-Effectiveness

The cost of combination therapy like DAP-ceftaroline is around 10 times more than monotherapy with VAN. But, if combination treatment can reduce the duration of bacteremia, it might be more economical than persistent MRSA with VAN as the latter can increase the duration of hospitalization. Combination treatment can also be de-escalated to monotherapy with more cost-effective drugs after bacterial clearance is achieved [61].

## Conclusions

Treatment of methicillin-resistant *Staphylococcus* bacteremia is a growing challenge that physicians continue to face as it can lead to life-threatening conditions. Although IDSA has recommended VAN and DAP as first-line treatment options for MRSA, multiple drawbacks warrant the advent of alternatives that will increase clinical success rates with fewer adverse effects. Many studies demonstrated that combining DAP/VAN with β-lactams can result in faster bacterial clearance and a lower risk of 30-day mortality - making it a promising choice. But these studies concerning ceftaroline were underpowered to detect clinically significant differences due to bias or smaller study groups. We need to conduct more head-to-head comparative studies with larger cohorts to replicate the results discussed here so that physicians can employ a comprehensive strategy against MRSA that will ensure increased clinical success with decreased mortality and morbidity, lower hospital stay, and reduced financial burden.
